# Prevalence of hepatitis C virus among patients with arthralgia: is it logic for screening?

**DOI:** 10.1186/s12985-023-02124-w

**Published:** 2023-07-21

**Authors:** Arwa Mohammed Othman, Asma’a Ahmed Al-hnhna, Belques Sharaf Al-Huraibi, Rowa Mohammed Assayaghi, Talal Yahya Al-Qahtani, Kamal Hamoud Jahzar, Marwan Mohammed Al-Huthaifi

**Affiliations:** 1 Microbiology and Immunology Department, Faculty of Medicine and Health Sciences, Sana’a University, Sana’a, Yemen; 2 Gastroenterology and Hepatology, Medicine department, 21 September University, Sana’a, Yemen; 3 Microbiology and Immunology Department, Faculty of Medicine and Health Sciences, Sana’a University, Sana’a-Yemen, Yemen; 4 Biomedical sciences department, Lebanese International University, Sana’a Compass, Yemen

**Keywords:** Arthralgia, Chronic hepatitis C, Hepatitis C virus, Extrahepatic manifestations

## Abstract

**Background:**

*Hepatitis C virus* (HCV) is well-known to be associated with multiple extrahepatic manifestations such as arthralgia, myalgia, arthritis, and vasculitis. Many studies reported frequent rheumatologic manifestations among patients infected by HCV. The purpose of this study was to determine the prevalence of HCV among chronic unexplained arthralgia patients in order to aid in the early detection and treatment of silent HCV infection.

**Methods:**

This study was a cross-sectional observational study conducted from July 2020 to May 2022. It included 145 individuals suffering from chronic unexplained arthralgia, with vast majority having oligoarticular joint pain (110, 75.9%). They were 103 (71%) females and 42 (29%) males. Serum samples from all patients were examined for the presence of anti-HCV antibodies using a rapid immunochromatographic assay. Seropositive samples were further examined using polymerase chain reaction (PCR) for detection of HCV RNA to confirm HCV infection.

**Results:**

Out of 145 patients who complained of arthralgia, seven patients tested positive for anti-HCV with a seroprevalence of 4.8% while five patients tested positive for HCV-RNA with a molecular prevalence of 3.4%. All positive patients were males (11.9%) with high statistical significance (**χ**^**2**^ = 12.7 and *p* = 0.002). No association was found between HCV infection and age, blood transfusion, surgery, using personal shaving tools, or being a health-care worker.

**Conclusions:**

The prevalence of HCV was high among males who complained of arthralgia. Patients with arthralgia, especially male patients, are recommended to perform HCV screening test.

## Background

Arthralgia is defined as pain in one or more joints, with no inflammation. There are many causes of arthralgia including injuries, infections, arthritis, and other disorders [1]. Many acute and chronic viral infections, such as Chikungunya and hepatitis B, are associated with polyarthralgias [2, 3]. Many studies reported arthralgia to be commonly associated with infection by hepatitis C virus [4–8].

*Hepatitis C virus* (HCV) is an enveloped, positive-sense single-stranded RNA virus belonging to the *Flaviviridae* family. Currently, HCV is classified into eight genotypes [9]. The global prevalence of HCV was estimated at 2.5%, ranging from 1.3% in Americas to 2.9% in Africa with significant morbidity and mortality [10]. HCV is a blood-borne virus that can be transmitted from person to person via exposure to contaminated blood such as through sharing drug-injection equipment, infants born to infected mothers, blood transfusion, sharing personal items (e.g., glucose monitors, razors, nail clippers, and toothbrushes). Though sexual transmission is uncommon, it has been reported more frequently among homosexual men [11]. In Yemen, the seroprevalence of HCV among general population was estimated to be 1.3%. However, seroprevalence among high risk groups was found to be: 1.5% in healthcare workers, 8.5% in pregnant women, and 40-62.7% in hemodialysis patients [12–15].

Less than 15% of people infected with HCV develop acute hepatitis while the remainder progress to chronic hepatitis. Chronic hepatitis C (CHC) can cause severe complications such as liver cirrhosis, hepatocellular carcinoma, and end-stage liver disease over the subsequent 30 years. Liver cirrhosis due to viral hepatitis C is the leading cause of liver transplantation in Europe [16–18]. HCV is not only associated with liver disease but is also frequently associated with multiple extrahepatic manifestations such as arthralgia, myalgia, arthritis, vasculitis, nephropathy, Sicca syndrome, and non-Hodgkin B-cell lymphoma [19, 20].

Arthralgia is the most common extrahepatic manifestation of CHC. In a study conducted in Brazil, arthralgia was found in 90.6% of patients infected with HCV [3]. Arthralgia is bilaterally symmetrical and involves more often the fingers, knee, and back. Arthralgia associated with CHC may mimic symptoms of rheumatoid arthritis. However, HCV-infected patients do not develop anticyclic citrullinated peptide antibodies (anti-CCP) which is helpful to distinguish between the two diseases [7].

Screening patients with arthralgia for HCV may be useful for HCV diagnosis and early treatment before progression to liver cirrhosis. Therefore, this study aimed to determine the prevalence of HCV among patients with chronic unexplained arthralgia.

## Methods

### Study design and area

This study was an observational cross-sectional study conducted from July 2020 to May 2022. Patients were attending Al-Thawrah Modern General Hospital, Al-Jomhori Educational Hospital, and Al-Kuwait University Hospital. All participants were investigated by Rheumatologists.

### Study population

A total of 145 patients who complained of chronic unexplained arthralgia were enrolled in this study. All participants aged 30 years and up with a mean age and standard deviation of 45 ± 12.6 years old. The study participants were 103 (71%) females and 42 (29%) males.

#### Inclusion criteria

Patients who complained arthralgia had no evidence of joint destruction on X-ray, normal liver function tests, and negative for both serum anti-CCP and cryoglobulins were enrolled.

#### Exclusion criteria

Patients who had arthritis, synovitis, mixed cryoglobulinemia vasculitis, abnormal liver function tests, serum anti-CCP positive, or those who had previously being diagnosed as HCV or hepatitis B virus positive.

### Sample size

The sample size was calculated using Epi Info version 6.04. Based on a population of 1,000,000 and the prevalence of arthralgia among patients infected with HCV of 8.8% in Egyptian study, the sample size at a 95% confidence level was 123 patients [21]. However, for more accuracy, 145 patients were enrolled in the current study.

### Data collection

Data such as age, gender, history of blood transfusion, or surgery were collected from each participant using a predesigned questionnaire. All patients gave informed consent forms prior to participation.

#### Specimen collection

After receiving informed consent, 3 ml of venous blood specimens were collected from each participant into plain tubes. Blood specimens were allowed to clot at room temperature. Clotted blood samples were centrifuged at 3000 rpm for 10 min. Serum was divided into two Eppendorf tubes; one for serological testing and the other for RT-PCR testing.

#### Determination of HCV infection

The diagnosis of hepatitis C virus among patients with arthralgia was based on the detection of anti-HCV antibodies in the patients’ serum using immunochromatographic device according to manufacturer’s instructions (Abon, USA). Sero-opsitive specimens were confirmed using real time polymerase chain reaction (RT-PCR) technique according to manufacture instructions (Sacace Biotechnologies, Italy).

### Statistical analysis

Data analysis was done using SPSS program version 20 (SPSS Inc., Chicago, IL, USA). Descriptive measures (mean ± standard deviation) were used for quantitative variables. Frequencies and percentages were used to present qualitative variables. Chi-square test was used for verifying existence of associations. P values ≤ 0.05 were considered statistically significant.

## Results

A total of 145 individuals who complained of arthralgia were examined for being infected with HCV. The study included 103 (71%) females and 42 (29%) males. Their ages ranged from 30 to 87 years, with a mean of 45.5 years and a standard deviation of 12.6 years. All patients had bilateral symmetrical joint pain and more than three-quarters (75.9%) of patients had oligoarticular joint pain, Table 1. The majority of patients 119 (82.1%) complained arthralgia for more than one year. All HCV infected patients were males with mean age 48.1 and a standard deviation of 7.9 years.

**Table 1:** Demographic and number of affected joints serologic tests of patients

	Total number of patients (145)	
	No.	%
**Gender**		
Males	42	29
Females	103	71
Age ± SD	45.5 ± 12.6 years	
**Number of affected joints**		
Monoarticular	29	20
Oligoarticular	110	75.9
Polyarticular	6	4.1

Seven (4.8%) patients with arthralgia were positive for HCV antibodies in their serum using a rapid immunochromatographic assay. Out of seven seropositive patients, five (3.4%) patients were confirmed to be positive for HCV-RNA using RT-PCR, Table 2.

**Table 2:** Sero- and molecular prevalence of HCV among patients complained arthralgia

	HCV positive		HCV negative	
	No.	%	No.	%
Immunochromatographic assay	7	4.8	138	95.2
RT-PCR	5	3.4	140	96.6

The inflammatory markers: CRP was positive in half of the patients (73, 50.3%) and ESR was high in 76 (52.4%) of patients. Two (28.6%) HCV-infected patients had positive CRP test whereas 3 (42.9%) HCV-infected patients had elevated ESR test. RF test, a non-specific test for RA, was positive in 17 (11.7%) patients while all patients were negative for the anti-CCP test which is specific for RA. Nevertheless, only one person (14.3%) of the patients infected with HCV had positive RF test, Table 3. Six (85.7%) of the HCV infected patients suffered from arthralgia for longer than one year and one (14.3%) for about seven months. All HCV infected patients had oligoarticular joint pain, however, none of them complained morning stiffness.

**Table 3:** Inflammatory markers test results of patients complained arthralgia

	Patients with negative HCV (N = 138)		HCV infected patients (N = 7)		Total number (145)	
Test	Number	%	Number	%	Number	%
**CRP test**						
Positive	71	51.4	2	28.6	73	50.3
Negative	67	48.6	5	71.4	72	49.7
**ESR test**						
High	73	52.9	3	42.9	76	52.4
Normal	65	47.1	4	57.1	69	47.6
**RF test**						
Positive	16	11.6	1	14.3	17	11.7
Negative	122	88.4	6	85.7	128	88.3

Table 4 shows some risk factors for HCV transmission among the study population who complained of arthralgia. All five HCV-positive patients were males but no HCV infection was found among females. This gender difference was statistically significant (χ^2^ = 12.7 and *P* = 0.002). All HCV infected patients belonged to age group 30–60 with no statistically significant difference (χ^2^ = 0.6 and *P* = 1). All HCV-infected patients had no history of surgery, blood transfusion or jaundice. Although all seropositive HCV patients did not use personal shaving tools, no significant statistical association was found (χ^2^ = 2.2 and *P* = 0.32). Two (7.4%) HCV-infected patients were healthcare workers while three (2.5%) patients were non-healthcare workers with no statistically significant difference (χ^2^ = 1.6 and *P* = 0.23).

**Table 4:** Potential risk factors for HCV transmission

	HCV positive		HCV negative		χ^2^	*p* ^*^
	No.	%	No.	%		
**Gender**						
Male	5	11.9	37	88.1	12.7	0.002
Females	0	0	103	100		
**Age groups**						
30–60	5	3.8	125	96.2	0.6	1
61–87	0	0	15	100		
**Surgery**						
Yes	0	0	59	100	3.5	0.08
No	5	5.8	81	94.2		
**Blood transfusion**						
Yes	0	0	23	100	0.98	1
No	5	4.1	117	95.9		
**History of jaundice**						
Yes	0	0	25	100	1.1	0.59
No	5	4.2	115	95.8		
**Using personal shaving tools**						
Yes	0	0	43	100	2.2	0.32
No	5	4.9	97	95.1		
**Health care workers**						
Yes	2	7.4	25	92.6	1.6	0.23
No	3	2.5	115	97.5		

*Fisher’s exact test

## Discussion

Arthralgia refers to pain in one or more of the body joints without inflammation. There are many different causes of arthralgia, such as injuries or infections [1]. HCV infection is responsible for both hepatic and extrahepatic manifestations. The spectrum of extra-hepatic manifestations varies from mild to moderate manifestations, for instance, arthralgia, Sicca syndrome, and peripheral neuropathies. Therefore, this observational cross-sectional study aimed to assess the prevalence of HCV among patients complained of arthralgia.

Our study revealed that the seroprevalence of HCV among patients complaining of arthralgia to be 4.8% while confirmed HCV infection using the PCR technique to be 3.4%. Many studies reported arthralgia to be the most common extrahepatic manifestations in HCV infection [20, 22, 23]. The mechanism through which chronic HCV infection causes arthralgia remains unclear. However, arthralgia could be attributed to formation of immune complexes due to continuous stimulation of the immune system by HCV proteins and to mixed cryoglobulinaemia which are reported to frequently occur in patients with chronic hepatitis C [24, 25].

This study showed that 17 (11.7%) arthralgia patients had positive RF test results while only one (14.3%) of the HCV-infected individuals was positive for RF test. This could be due to that chronic stimulation of B lymphocytes by HCV directly changes the function of B and T lymphocyte resulting in polyclonal activation and expansion of B- cell-producing antibodies that have RF activity [26].

All HCV- positive patients were males whereas no HCV infection was detected among females. Although Gacche and Al-Mohani, 2012, reported prevalence of HCV to be higher among Yemeni females, the higher frequency of HCV infection among males complained of arthralgia in this study might be related to the higher ability of females to clear HCV as compared to males [12, 27].

This study showed no significant association between HCV infection and blood transfusion. This could be explained by the fact that the risk of HCV transmission through blood transfusion is now rare because blood donors are investigated for presence of HCV in their blood before blood donation.

This study showed no significant association between HCV infection and the history of surgery which may be attributed to effective and adequate sterilization of surgical instruments. Two out of five HCV patients were nurses, despite no significant association, this might be due to higher exposure to needle stick injury among nurses.

### Conclusion and recommendation

In conclusion, HCV prevalence was more frequent among males suffering arthralgia than females, thereby, we recommended HCV screening test for patients complaining of arthralgia especially male patients which may help in the early diagnosis of a silent infection and subsequently avoidance liver cirrhosis and hepatocellular carcinoma.

## Data Availability

The data that support the findings of this study are available. Anyone interested can get upon reasonable request from corresponding author.
